# The High Stability and Selectivity of Electrochemical Sensor Using Low-Cost Diamond Nanoparticles for the Detection of Anti-Cancer Drug Flutamide in Environmental Samples

**DOI:** 10.3390/s24030985

**Published:** 2024-02-02

**Authors:** Nareshkumar Baskaran, Sanjay Ballur Prasanna, Yu-Chien Lin, Yeh-Fang Duann, Ren-Jei Chung, Yang Wei

**Affiliations:** 1 Department of Chemical Engineering and Biotechnology, National Taipei University of Technology, Taipei 10608, Taiwan; adamnaresh1818@gmail.com (N.B.); sanjaypgowda43@gmail.com (S.B.P.); yuchien.lin91@gmail.com (Y.-C.L.); f10421@mail.ntut.edu.tw (Y.-F.D.); 2 School of Materials Science and Engineering, Nanyang Technological University, Singapore 639798, Singapore; 3 High-Value Biomaterials Research and Commercialization Center, National Taipei University of Technology, Taipei 10608, Taiwan

**Keywords:** flutamide, diamond, anti-cancer drug, environmental samples, screen-printed carbon electrode

## Abstract

In this study, a novel electrochemical sensor was created by fabricating a screen-printed carbon electrode with diamond nanoparticles (DNPs/SPCE). The successful development of the sensor enabled the specific detection of the anti-cancer drug flutamide (FLT). The DNPs/SPCE demonstrated excellent conductivity, remarkable electrocatalytic activity, and swift electron transfer, all of which contribute to the advantageous monitoring of FLT. These qualities are critical for monitoring FLT levels in environmental samples. Various structural and morphological characterization techniques were employed to validate the formation of the DNPs. Remarkably, the electrochemical sensor demonstrated a wide linear response range (0.025 to 606.65 μM). Additionally, it showed a low limit of detection (0.023 μM) and high sensitivity (0.403 μA μM^−1^ cm^−2^). Furthermore, the practicability of DNPs/SPCE can be successfully employed in FLT monitoring in water bodies (pond water and river water samples) with satisfactory recoveries.

## 1. Introduction

Flutamide (FLT), also known by its scientific name 4-nitro-3-trifluoromethyl-isobutilanilide, is an oral non-steroidal anti-androgen drug that is commonly utilized to control and treat patients suffering from prostate cancer [1,2]. Initially, FLT was utilized as an anti-bacterial agent, which was later found to contain the properties of an anti-androgenic drug. Most of the anti-androgen drugs effectively treat patients, specifically men with prostate cancer, by controlling the release of the male sex hormone, testosterone, which is the most important source for the growth and proliferation of prostate cancer cells. Thus, FLT does not completely cure prostate cancer; however, it helps in prolonging the survival of patients suffering from prostate cancer for several months or years by keeping the cancer cells under static conditions through the effective inhibition of testosterone binding to prostate tissues [3,4]. Furthermore, FLT has the potential to treat female patients suffering from excess androgen levels in their body due to polycystic ovarian syndrome (PCOS) [5,6]. However, like any medication, an overdosage of this FLT drug may cause numerous side effects in addition to the expected effects; some of the adverse side effects include diarrhea, nausea, an inflamed prostate, rectal bleeding, loss of sexual interest, blood in urine, liver malfunction, drowsiness, and methemoglobinemia. In addition, the transfer of FLT drugs to river water via effluents from waste-water treatment plants has been reported and the samples recovered from those environmental sources have confirmed the presence of FLT from varying concentration ranges of nanograms to even a few milligrams per liter [2,7,8]. Creatures living in this environment or consuming the FLT-polluted water will have adverse side effects.

Various conventional techniques and methodologies, including high-performance liquid chromatography (HPLC), spectrophotometry, flow injection and fluorescence analysis, polarography, and gas–liquid chromatography (GLC), have been utilized for the identification of FLT [9,10,11,12]. While many techniques exhibit outstanding sensitivity, they are burdened by notable limitations including a time-consuming sample pretreatment process, expensive equipment with high operational costs, and the necessity for highly skilled technicians. In recent years, electrochemical analysis has preferred attraction and attention compared to conventional techniques due to their low cost, high sensitivity, rapid response, selectivity, simplicity, and portability. Moreover, they have shown a good ability in detecting the preferred analytes in real samples. Hence, constructing a facile and convenient electrochemical sensor will be advantageous for the detection of FLT.

To enhance the performance of the working electrode with better sensitivity and selectivity, it must be modified with some conductive materials. Some of the commonly used carbon and carbon-based nanomaterials are graphene and its oxide, carbon nanotubes, such as MWCNTs (multiwalled carbon nanotubes), and carbon quantum dots. These materials have a few drawbacks including extensive and complicated synthesis processes, a high cost, and sometimes poor electrocatalytic performance. Recently, diamond nanoparticles (DNPs) as a carbon-based nanomaterial have gained appreciable attention for their excellent biocompatibility, moderate price, non-cytotoxic nature, easy handling, and narrow size distribution [13]. Nevertheless, the usage of DNPs for electrochemical studies is still scarce due to their higher band gap value (5.47 eV), which gives them an insulating property, which is less attractive for electrochemical applications when compared to other carbon materials. Despite this drawback, several fundamental studies have reported DNPs as one of the emerging nanomaterials with most potential for the fabrication of electrode surfaces due to their high electrochemical response towards various redox probes in solutions of Fe(CN_6_)^3−/4−^, Ru(NH_3_)_6_^2+/3+^, IrCl_6_^2−/3−^, and Ru(CN)_6_^3−/4−^, which is related to the presence of functional groups with one or more unsaturated bond on the diamond nanoparticle’s surface [14,15,16,17,18]. Thus, although DNPs show non-conducting behavior in a bulk state, their characteristics might shift at the nanoscale due to some unexpected phenomena, such as displaying redox activity. 

In this work, we utilized undoped DNPs as an electrode modifier for the selective detection of FLT. By incorporating DNPs, the SPCE was effectively modified to exhibit exceptional sensitivity toward the FLT. The enhanced electrocatalytic properties of the DNPs played a crucial role in achieving this performance. The modified SPCE demonstrated excellent detection capabilities within a linear range of 0.025 to 605.65 µM, surpassing expectations with a low detection limit of 0.023 µM. This sensor exhibits excellent conductivity, superior electrocatalytic activity, and remarkable electron transfer capability, rendering it highly efficient in the monitoring of FLT. Hence, it is crucial to identify and oversee the FLT medication in various environmental applications.

## 2. Materials and Methods

### 2.1. Material

Sigma-Aldrich was the source for all reagents, such as diamond nanopowder (<10 nm), ethanol (C_2_H_5_OH), and FLT. No purification process was carried out on any of the materials used. Throughout the experiments, deionized (DI) water was employed for the preparation of solutions and washing purposes. The TE-100 model of screen-printed carbon electrodes (SPCEs) was acquired from Zensor (Anchorage, AL, USA) for this study.

### 2.2. Instruments

The crystallinity in DNPs was detected using an X-ray powder diffractometer (XRD, X′Pert3 Powder, PANalytical, Almelo, The Netherlands). Raman spectra were measured on the Ramboss 500i Micro (DINGWOO/USA). Field-emission scanning electron microscope (FESEM/JEOL JSM-7100F) was employed for investigating the morphology of the DNPs. The electrochemical studies were carried out using a three-electrode system using an electrochemical workstation CH Instruments 6041E workstation (Shanghai Chenhua, Shanghai, China). The working electrode employed in this study was a screen-printed carbon electrode (SPCE) with dimensions of 3 mm/0.071 cm^2^. A silver/silver chloride (Ag/AgCl) electrode was used as the reference electrode, while a platinum wire served as the counter electrode.

### 2.3. Fabrication of Modified Electrodes

Before modification, the SPCEs underwent a thorough cleaning process using deionized water and were subsequently dried at a temperature of 50 ℃ in an oven until completely dry. The dispersion process involved dispersing 2 mg of DNPs into 1 mL of DI water, followed by ultrasonication for 30 min. Afterward, the suspension was utilized to drop cast 4 μL onto the pre-treated SPCE surface, which was subsequently dried at 50 °C. This procedure yielded the DNPs-modified SPCE.

### 2.4. Collection of Environmental Samples

The collection of water samples involved obtaining pond water from the NTUT campus and river water from the Xindian River in northern Taiwan. Centrifugation of the water samples was carried out at 9000 rpm for 15 min. Subsequently, the supernatant was discarded, and the samples were stored at a temperature of 4 °C for further analysis in real sample testing.

## 3. Results

### 3.1. Characterizations

**Figure 1:**
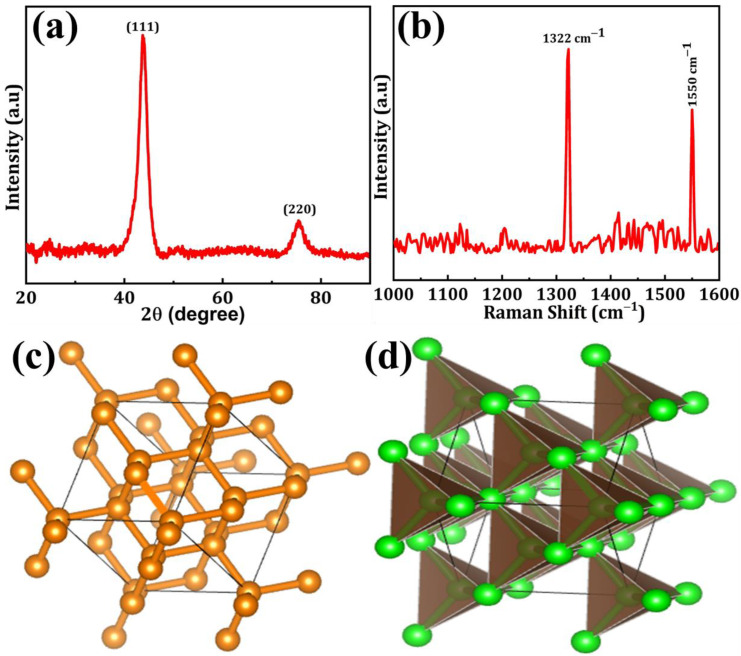
(**a**) XRD patterns, (**b**) Raman spectra, (**c**) ball-stick, and (**d**) polyhedron structure of DNPs.

The XRD spectra of DNPs are depicted in Figure 1a. The XRD pattern exhibits a dominant peak positioned at 43.6° and a secondary peak positioned at 75.5°, representing the crystal planes (111) and (220) of DNPs, respectively [19]. Figure 1b displays the Raman spectra of DNPs. The sharp and intense peak observed in the corrected spectra, obtained by plotting the Raman intensity from 1000 to 1650 cm^−1^, corresponds to the first-order DNPs peak located at approximately 1332 cm^−1^. This peak corresponds to the vibrational mode involving the stretching and compression of the carbon-carbon (C–C) bonds in the DNPs lattice. Furthermore, the emergence of the G-band peak at approximately 1550 cm^−1^ can be observed, indicating the presence of in-plane stretching of sp2 bonds within the graphite-like substances [20]. The possible ball-stick and polyhedron structures of DNPs are shown in Figure 1c,d.

**Figure 2:**
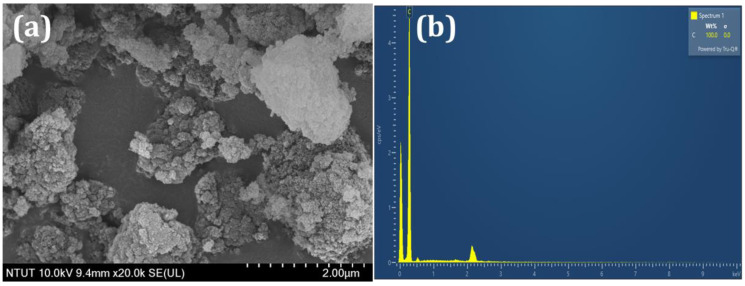
(**a**) SEM image and (**b**) EDX analysis of DNPs.

SEM analysis was utilized to investigate the surface morphology of the DNPs. By utilizing typical SEM micrographs, Figure 2a presents the visual aspect of the DNPs, revealing their agglomerated particle-like structure. The presence of carbon was confirmed by the EDX spectrum shown in Figure 2b. The SEM results confirmed the presence of a DNPs structure, as indicated by the obtained results.

### 3.2. Electrochemical Performances

**Figure 3:**
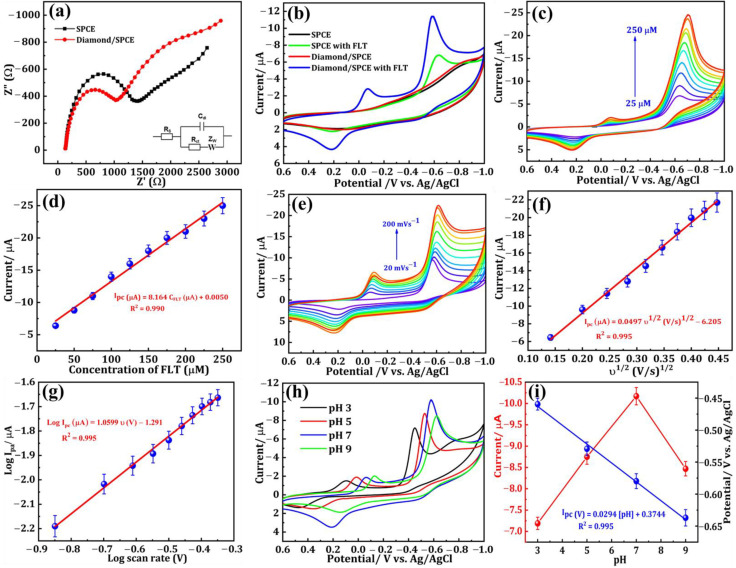
(**a**) EIS spectra of SPCE (Black dots) and DNPs-modified SPCE (Red dots) in 0.05 M [Fe (CN)_6_]^3−/4−^ containing 0.1 M KCl (Inset: Randles equivalent circuit). (**b**) CV response of SPCE (with and without FLT), and DNPs/SPCE in 75 µM of FLT containing 0.1 M PBS (pH = 7.0). (**c**) CV responses with increasing FLT concentration (25 to 200 µM). (**d**) I_pc_ vs. square root of the scan rate. (**e**) CV curves for scan rates (20 to 200 mVs^−1^) in 75 µM FLT (0.1 M, pH 7.0). (**f**) I_pc_ vs. square root of scan rate and (**g**) Log I_pc_ vs. log v. (**h**) CV curves of FLT (75 µM) at diverse pH (3, 5, 7, and 9). (**i**) values of I_pc_ and E_p_ versus pH.

The measurement technique known as electrochemical impedance spectroscopy (EIS) is extensively preferred for examining the electron transfer capability of modified electrodes. The diameter of the semicircle represents the charge transfer resistance (R_ct_) at high frequencies, while the linear portion characterizes the diffusion process at low frequencies. The EIS spectra of the SPCE and the DNPs-modified SPCE, conducted at a potential of 0.27 V in a solution containing 0.05 M [Fe(CN)_6_]^3−/4−^ and 0.1 M KCl, are depicted in Figure 3a. The frequency range for the spectra analysis was set from 1 Hz to 100 kHz. By computing the diameter of the obtained semicircle, the R_ct_ value was successfully determined. The SPCE exhibited a semicircle with an Rct value of 1255.25 Ω, which can be attributed to its elevated resistance. On the other hand, when the SPCE was modified with DNPs it exhibited a smaller semicircle compared with the SPCE. From this EIS data, we could see that modifying the SPCE electrode with DNPs increased its conductivity through there being more available active sites, making the DNPs-modified SPCE an excellent material for the sensing applications toward FLT.

The CV technique was employed to analyze the electrochemical behavior of modified electrodes in a nitrogen gas-saturated 0.1 M phosphate buffer solution (PBS, pH 7.0). The reduction of FLT was specifically investigated within a fixed scan rate of 50 mVs^−1^ and a potential range of +0.6 to −1.0 V. Figure 3b displays the CVs of the SPCE and DNPs-modified SPCE without FLT, and the SPCE and DNPs-modified SPCE containing 0.01 M of FLT in 0.1 M PBS. In Figure 3b, we can see that the curve obtained for SPCE and DNPs/SPCE showed no significant peak in the absence of 0.01 M of FLT. In contrast, the DNPs-modified electrode exhibited a distinct and sharp cathodic peak at a lower potential of −0.58 V upon the addition of 0.01 M FLT to the 0.1 M PBS electrolyte solution. The emergence of this peak can be attributed to the complete reduction of FLT to hydroxylamine. The cathodic current peak observed in this experiment was deemed irreversible as there was no corresponding anodic oxidation peak current detected. 

**Figure 4:**
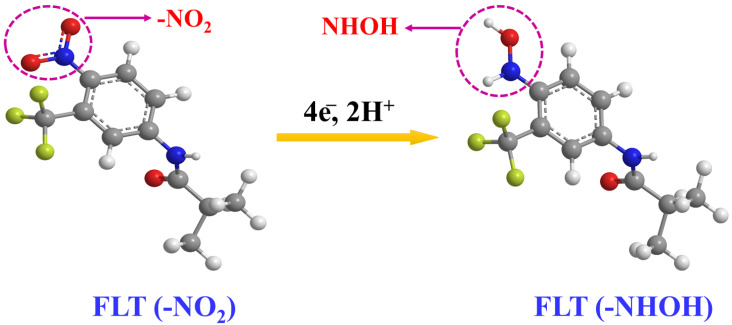
The possible electro-reduction of flutamide.

Additionally, we could observe the appearance of two more reversible peaks in the DNPs-modified SPCE in the presence of 0.01 M FLT. The potential of 0.22 V revealed an anodic peak, while the peak observed at a potential of −0.06 V corresponds to its cathodic peak. These peaks appeared because of a two-electron reversible reaction between a hydroxylamine derivative and a nitroso derivative. The reduction of the nitro group in FLT resulted in the emergence of a cathodic peak observed at a potential of −0.06 V, indicating the formation of hydroxylamine derivatives [2]. The electrochemical mechanism of FLT has been convincingly illustrated in Figure 4. Additionally, the DNPs-modified SPCE exhibited a cathodic reduction peak current response that was approximately two times greater than the SPCE in the presence of 0.01 M FLT. The findings strongly indicate that the SPCE electrode, modified with DNPs, demonstrates exceptional electrocatalytic activity in detecting FLT.

Figure 3c displays the results of the analysis conducted to evaluate the electrochemical performance of the DNPs-modified SPCE in detecting different concentrations of FLT. Cyclic voltammetry (CV) was employed in a 0.1 M PBS solution with a pH of 7.0, utilizing a scan rate of 50 mVs^−1^. The peak current density was observed to increase when the concentration of FLT was raised from 25 μM to 250 μM. This enhancement can be attributed to the reduction of FLT at the surface of the DNPs-modified electrode, highlighting the sensitivity of FLT towards the DNPs-modified SPCE. Moreover, from the calibration plot provided in Figure 3d, we could see a good linear relationship between the different concentrations of FLT and reduction peak current with a correlation coefficient (R^2^) of 0.990. The potential of the DNPs-modified SPCE as a modified electrode for FLT detection was demonstrated. The resulting linear regression equation was derived (Equation (1)):

I_pc_ (μA) = 8.164 C_FLT_ (μM) + 0.005 (R^2^ = 0.990) (1)

The performance of a modified electrode is also affected by the scan rate, which was investigated by varying the scan rate using the CV technique. The CV curves of the DNPs-modified electrode in 0.01 M FLT with a scan rate ranging from 20 to 200 mV s^−1^ in 0.1 M PBS (pH 7.0) are depicted in Figure 3e. We could see that there is a clear increase in the peak current density with the increase in scan rate. On fitting the reduction peak current density against the varying scan rate, a linear relationship was obtained, which can be seen in Figure 3f. The log current vs. log v is shown in Figure 3g. From the obtained scan rate results, the electrochemical reduction of FLT on the DNPs-modified SPCE was a diffusion-controlled reaction [21]. The resulting linear regression equation was derived (Equations (2) and (3)):

I_pc_ (μA) = 0.0497 υ^1/2^ (V/s)^1/2^ − 6.205 (R^2^ = 0.995) (2)

Log I_pc_ (μA) = 1.0599 log υ (V) − 1.291 (R^2^ = 0.995) (3)

By utilizing CV analysis, the effect of the electrolyte’s pH (PBS) on the electrochemical efficiency of DNPs-modified SPCE in detecting 0.01 M FLT in 0.1 M PBS was thoroughly examined. The obtained results are presented in Figure 3h. The increase in pH from 3.0 to 7.0 resulted in a noticeable rise in the reduction peak current density value, followed by a decrease at pH 9.0. Furthermore, variations in the maximum potential can be observed across different pH levels, suggesting that the decrease in FLT is undeniably influenced by the pH of the electrolyte system employed. Furthermore, the findings suggest that the electrochemical reduction reaction of FLT involves the participation of both protons and electrons. The calibration plot in Figure 3i illustrates the relationship between the different pH versus peak potential and peak current, which were obtained from the electrolyte system at different pH levels. Throughout our studies, we employed the PBS electrolyte with a pH of 7.0 to achieve the highest reduction current peak, thereby enhancing the sensitive detection of FLT. The correlated regression equations are as follows (Equation (4))

E_pc_ (V) = 0.0294 [pH] + 0.3744 (R^2^ = 0.995) (4)

Utilizing the obtained slope (0.0294), the Nernst equation was applied to calculate $Ep=-\frac{0.0592m}{n}pH+b,$ enabling the determination of the m/n ratio [22]. The values of m and n represent the quantities of 2H^+^ and 4e^−^ engaged in the reduction of FLT, respectively. The calculated ratio of m/n is approximately 0.49. The findings suggest that both 2H^+^ and 4e^−^ play a role in the reaction [4].

### 3.3. DPV Performances

**Figure 5:**
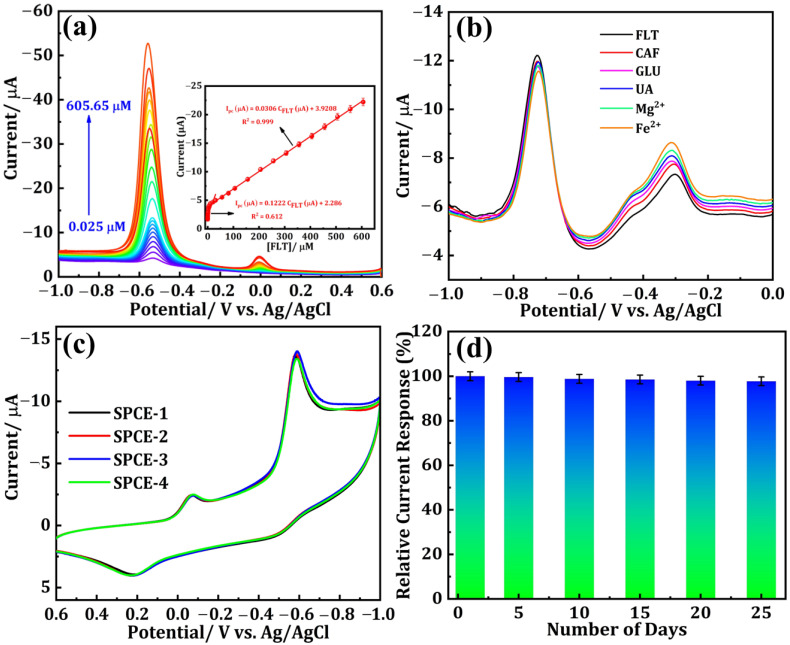
(**a**) DPV responses for the various concentrations of FLT from 0.025 to 605.65 μM using the DNPs-modified SPCE (Inset: the calibration plot between the peak current vs. FLT concentration). (**b**) DPV curves for the DNPs/SPCE in the presence of FLT and FLT with various interfering substances. (**c**) Reproducibility and (**d**) storage stability.

Differential pulse voltammetry (DPV) is widely acknowledged as a highly reputable technique renowned for its exceptional ability to detect analytes at trace-level concentrations with remarkable sensitivity. The current response of DNPs-modified SPCE with the successive additions of different concentrations of FLT in 0.1 M PBS (pH 7.0) is illustrated in Figure 5a. The cathodic peak current density exhibits a gradual increase as the concentration of FLT rises within a broad linear range of 0.025 to 605.65 μM. By analyzing the relationship between I_pc_ and FLT concentration (Figure 5a (inset)), two linear fits were determined. The achieved linearity ranges from 0.025 to 20.65 µM, with a calculated correlation coefficient (R^2^) of 0.999, and the related equation is shown below (Equation (5)).

I_pc_ (μA) = 0.1222 C_FLT_ (μM) + 2.286 (R^2^ = 0.612) (5)

In addition, second linearity was attained within the ranges of 30.65 to 605.65 µM, while the correlation coefficient (R^2^) was determined to be 0.999 and the related equation is shown below (Equation (6)).

I_pc_ (μA) = 0.0306 C_FLT_ (μM) + 3.9208 (R^2^ = 0.999) (6)

At low concentrations, the FLT molecules exhibit swift movement toward the surface of the SPCE. Consequently, an immediate reaction occurs as the FLT concentration rises. Nevertheless, subsequent increments in concentration considerably impede the movement of molecules. Two distinct linear regression fits were obtained due to the disparate response of I_pc_ [23]. The detection limit (LOD) for FLT was determined to be 0.023 µM. Hence, the suggested SPCE modified with DNPs holds great potential as an electrode material for the accurate detection of FLT, an anti-cancer drug. Moreover, while compared with other reported sensors, the proposed electrochemical sensor in our study shows a wider linear range of 0.025 to 605.65 µM, along with a lower detection limit of 0.023 µM (Table 1).

The selectivity of the DNPs/SPCE sensor for FLT reduction with diverse interferants was examined using DPV. Figure 5b shows the DPV curves of FLT-containing 0.1 M PBS (pH 7) with twofold concentrations of various interfering species including caffeine (CAF), glucose (GLU), uric acid (UA), magnesium chloride (Mg^2+^), and iron chloride (Fe^2+^). From the outcomes, the chosen interferants did not interfere with the FLT detection. Consequently, the designed sensor exhibited higher selectivity for FLT detection. The reproducibility of the proposed DNPs-modified electrode, which is inevitable for the real-time monitoring of FLT, was studied by performing the CV technique with the addition of 0.01 M FLT in nitrogen gas-saturated 0.1 M PBS (pH 7.0) at a fixed scan rate of 50 mVs^−1^ and a potential range of +0.6 to −1.0 V on four different DNPs-modified SPCE (E1, E2, E3, and E4). The obtained results are shown in Figure 5c, from which all four independent electrodes modified using DNPs exhibited the same results with minimal fluctuation in the peak current value, indicating that the proposed electrode in our study has good reproducibility. The stability of the proposed sensor in our study was investigated by storing the DNPs-modified SPCE for 25 days and the current values were noted once at an interval of every five days, which are depicted as a bar graph in Figure 5d.

### 3.4. Real Sample Analysis in Environmental Samples

**Figure 6:**
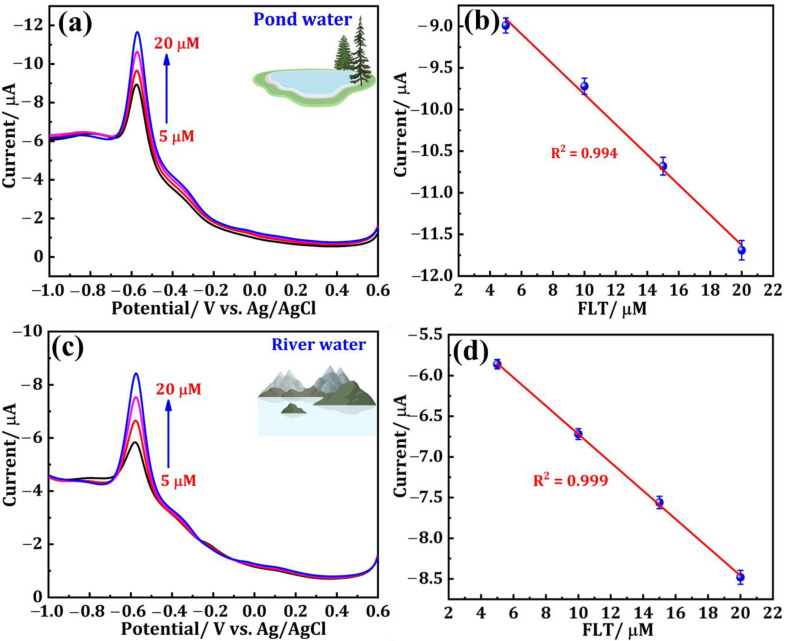
DPV response of (**a**) pond water and (**c**) river water samples containing FLT at different concentrations. (**b**,**d**) Corresponding linear plots.

The effectiveness of the DNPs/SPCE for FLT sensing was examined in water bodies, specifically pond water and river water samples. The pretreatment steps for the water samples are elucidated in Section 2.4. Before experimenting, each of the two different samples was diluted with 9 mL of PBS (pH = 7.0) separately, resulting in a total volume of 10 mL. These diluted samples were then utilized as the supporting electrolyte. The DPV method was employed to record the electrochemical signal of the real samples containing supporting electrolytes. Sequential addition of FLT (5–20 μM), with a known concentration of 0.001 M, was carried out during the experiment and the consistent results are displayed in Figure 6a–d. Figure 6a depicts the DPV response of a pond water sample containing FLT at concentrations of 5, 10, 15, and 20 µM. Figure 6b shows the corresponding linear plot is also presented. Similarly, the DPV curves of FLT (5, 10, 15, 20 µM)-spiked river samples and the corresponding linear plots are presented in Figure 6c,d, respectively. The calculations were performed to determine the recoveries of the two distinct samples, and the outcomes are documented in Table 2. The data obtained from this study strongly suggest that the DNPs/SPCE exhibits a substantial ability to detect FLT in different water bodies.

## 4. Conclusions

In summary, for the first time, we achieved excellent selective and sensitive electrochemical detection of the anti-cancer drug FLT using a DNPs-modified SPCE. DNPs have exhibited excellent electrocatalytic activity at the nanoscale, overcoming the insulating character, which was determined using electrochemical impedance spectroscopy (EIS), cyclic voltammetry (CV), and difference pulse voltammetry (DPV) techniques. The DNPs-modified SPCE employed as an electrochemical sensor exhibited excellent sensitivity, and good stability and selectivity towards the detection of FLT with repeatability and reproducibility. Moreover, it provided a wide linear response range (0.025 µM to 605.65 µM) and low detection limit (0.023 µM). These obtained results indicate that the DNPs can be used as an alternative carbon-based electrode modification material for the selective determination of FLT. Furthermore, the practicability of DNPs/SPCE can be successfully employed in FLT monitoring in water bodies (pond water and river water samples) with satisfactory recoveries.

## Figures and Tables

**Table 1:** Electroanalytical performance of FLT using DNPs/SPCE and other electrochemical sensors.

Electrode Material	Method	pH	Linear Range (μM)	LOD (μM)	References
Zn-TIT_4_A-L@RGO	DPV	6	0.1–200	0.015	[24]
MoW-P/RGO	DPV	7	0.3–1152	0.009	[25]
MXene/MOF	DPV	7	0.05–70	0.015	[26]
MXene/MIL-101(Cr)	DPV	7	0.025–100	0.009	[27]
SF-CTAB	DPV	7	0.016–658.51	0.007	[28]
DNPs/SPCE	DPV	7	0.025–605.65	0.023	Present Work

**Table 2:** Determination of FLT in pond and river water samples using the DNPs/SPCE sensor.

Samples	Added (µM)	Found (µM)	Recovery (%)	RSD (%)
Pond water	5	4.72	94.4	1.8
	10	9.92	99.2	1.7
	15	14.79	98.6	1.9
	20	19.2	96.0	2.1
River water	5	4.89	97.8	2.4
	10	9.15	91.5	2.6
	15	14.33	95.5	2.1
	20	19.63	98.15	1.8

## Data Availability

Data are contained within the article.
